# A narrative review on the validity of electronic health record-based research in epidemiology

**DOI:** 10.1186/s12874-021-01416-5

**Published:** 2021-10-27

**Authors:** Milena A. Gianfrancesco, Neal D. Goldstein

**Affiliations:** 1 Division of Rheumatology, University of California School of Medicine, San Francisco, CA USA; 2 Department of Epidemiology and Biostatistics, Drexel University Dornsife School of Public Health, 3215 Market St., Philadelphia, PA 19104 USA

**Keywords:** Electronic health records, Validity, Bias, Data quality, Secondary analysis

## Abstract

Electronic health records (EHRs) are widely used in epidemiological research, but the validity of the results is dependent upon the assumptions made about the healthcare system, the patient, and the provider. In this review, we identify four overarching challenges in using EHR-based data for epidemiological analysis, with a particular emphasis on threats to validity. These challenges include representativeness of the EHR to a target population, the availability and interpretability of clinical and non-clinical data, and missing data at both the variable and observation levels. Each challenge reveals layers of assumptions that the epidemiologist is required to make, from the point of patient entry into the healthcare system, to the provider documenting the results of the clinical exam and follow-up of the patient longitudinally; all with the potential to bias the results of analysis of these data. Understanding the extent of as well as remediating potential biases requires a variety of methodological approaches, from traditional sensitivity analyses and validation studies, to newer techniques such as natural language processing. Beyond methods to address these challenges, it will remain crucial for epidemiologists to engage with clinicians and informaticians at their institutions to ensure data quality and accessibility by forming multidisciplinary teams around specific research projects.

## Background

The proliferation of electronic health records (EHRs) spurred on by federal government incentives over the past few decades has resulted in greater than an 80% adoption-rate at hospitals [1] and close to 90% in office-based practices [2] in the United States. A natural consequence of the availability of electronic health data is the conduct of research with these data, both observational and experimental [3], due to lower overhead costs and lower burden of study recruitment [4]. Indeed, a search on PubMed for publications indexed by the MeSH term “electronic health records” reveals an exponential growth in biomedical literature, especially over the last 10 years with an excess of 50,000 publications.

An emerging literature is beginning to recognize the many challenges that still lay ahead in using EHR data for epidemiological investigations. Researchers in Europe identified 13 potential sources of “bias” (bias was defined as a contamination of the data) in EHR-based data covering almost every aspect of care delivery, from selective entrance into the healthcare system, to variation in care and documentation practices, to identification and extraction of the right data for analysis [5]. Many of the identified contaminants are directly relevant to traditional epidemiological threats to validity [4]. Data quality has consistently been invoked as a central challenge in EHRs. From a qualitative perspective, healthcare workers have described challenges in the healthcare environment (e.g., heavy workload), imperfect clinical documentation practices, and concerns over data extraction and reporting tools, all of which would impact the quality of data in the EHR [6]. From a quantitative perspective, researchers have noted limited sensitivity of diagnostic codes in the EHR when relying on discrete codings, noting that upon a manual chart review free text fields often capture the missed information, motivating such techniques as natural language processing (NLP) [7]. A systematic review of EHR-based studies also identified data quality as an overarching barrier to the use of EHRs in managing the health of the community, i.e. “population health” [8]. Encouragingly this same review also identified more facilitators than barriers to the use of EHRs in public health, suggesting that opportunities outweigh the challenges. Shortreed et al. further explored these opportunities discussing how EHRs can enhance pragmatic trials, bring additional sophistication to observational studies, aid in predictive modeling, and be linked together to create more comprehensive views of patients’ health [9]. Yet, as Shortreed and others have noted, significant challenges still remain.

It is our intention with this narrative review to discuss some of these challenges in further detail. In particular, we focus on specific epidemiological threats to validity -- internal and external -- and how EHR-based epidemiological research in particular can exacerbate some of these threats. We note that while there is some overlap in the challenges we discuss with traditional paper-based medical record research that has occurred for decades, the scale and scope of an EHR-based study is often well beyond what was traditionally possible in the manual chart review era and our applied examples attempt to reflect this. We also describe existing and emerging approaches for remediating these potential biases as they arise. A summary of these challenges may be found in Table 1. Our review is grounded in the healthcare system in the United States, although we expect many of the issues we describe to be applicable regardless of locale; where necessary, we have flagged our comments as specific to the U.S.

**Table 1:** A summary of the challenges faced by epidemiologists when conducting electronic health record-based research, their manifestations in terms of threats to validity, and potential solutions

Challenge	Sub-challenge	Example	Threat(s) to validity	Potential solution(s)
#1 Representativeness	--	Catchment of a federally qualified health center versus academic medical center	Selection bias and generalizability	Comparison to external data; Inverse probability weighting for selection bias
#2 Data availability and interpretation	2.1 Billing versus Clinical versus Epidemiological Needs	Presence or absence of diagnostic codes	Information bias and confounding	Validation study; quantitative bias analysis
	2.2 Consistency in Data and Interpretation	Variations in reported laboratory results	Information bias and confounding	Validation study; quantitative bias analysis
	2.3 Unstructured Data: Clinical Notes and Reports	Operationalizing phenotypes from the encounter note	Information Bias and confounding	Natural language processing
#3 Missing measurements	--	Socioeconomic status not captured	Information or Selection Bias, Confounding	Imputation, Surrogate Measures, Validation Study
#4 Missing visits	--	Lack of longitudinal view of patient	Information or Selection Bias	Imputation, Surrogate Measures, Validation Study

## Challenge #1: Representativeness

The selection process for how patients are captured in the EHR is complex and a function of geographic, social, demographic, and economic determinants [10]. This can be termed the *catchment* of the EHR. For a patient record to appear in the EHR the patient must have been registered in the system, typically to capture their demographic and billing information, and upon a clinical visit, their health details. While this process is not new to clinical epidemiology, what tends to separate EHR-based records from traditional paper-based records is the scale and scope of the data. Patient data may be available for longer periods of time longitudinally, as well as have data corresponding to interactions with multiple, potentially disparate, healthcare systems [11]. Given the consolidation of healthcare [12] and aggregated views of multiple EHRs through health information networks or exchanges [11] the ability to have a complete view of the patients’ total health is increasing. Importantly, the epidemiologist must ascertain whether the population captured within the EHR or EHR-derived data is representative of the population targeted for inference. This is particularly true under the paradigm of population health and inferring the health status of a community from EHR-based records [13]. For example, a study of *Clostridium difficile* infection at an urban safety net hospital in Philadelphia, Pennsylvania demonstrated notable differences in risk factors in the hospital’s EHR compared to national surveillance data, suggesting how catchment can influence epidemiologic measures [14]. Even health-related data captured through health information exchanges may be incomplete [15].

Several hypothetical study settings can further help the epidemiologist appreciate the relationship between representativeness and validity in EHR research. In the first hypothetical, an EHR-based study is conducted from a single-location federally qualified health center, and in the second hypothetical, an EHR-based study is conducted from a large academic health system. Suppose both studies occur in the same geographic area. It is reasonable to believe the patient populations captured in both EHRs will be quite different and the catchment process could lead to divergent estimates of disease or risk factor prevalence. The large academic health system may be less likely to capture primary care visits, as specialty care may drive the preponderance of patient encounters. However, this is not a bias *per se*: if the target of inference from these two hypothetical EHR-based studies is the local community, then selection bias becomes a distinct possibility. The epidemiologist must also consider the potential for generalizability and transportability -- two facets of external validity that respectively relate to the extrapolation of study findings to the source population or a different population altogether -- if there are unmeasured effect modifiers, treatment interference, or compound treatments in the community targeted for inference [16].

There are several approaches for ascertaining representativeness of EHR-based data. Comparing the EHR-derived sample to Census estimates of demography is straightforward but has several important limitations. First, as previously described, the catchment process may be driven by discordant geographical areas, especially for specialty care settings. Second and third, the EHR may have limited or inaccurate information on socioeconomic status, race, and ethnicity that one may wish to compare [17, 18], and conversely the Census has limited estimates of health, chiefly disability, fertility, and insurance and payments [19]. If selection bias is suspected as a result of missing visits in a longitudinal study [20] or the catchment process in a cross-sectional study [21], using inverse probability weighting may remediate its influence. Comparing the weighted estimates to the original, non-weighted estimates provides insight into differences in the study participants. In the population health paradigm whereby the EHR is used as a surveillance tool to identify community health disparities [13], one also needs to be concerned about representativeness. There are emerging approaches for producing such small area community estimates from large observational datasets [22, 23]. Conceivably, these approaches may also be useful for identifying issues of representativeness, for example by comparing stratified estimates across sociodemographic or other factors that may relate to catchment. Approaches for issues concerning representativeness specifically as it applies to external validity may be found in these references [24, 25].

## Challenge #2: Data availability and interpretation

### Sub-challenge #2.1: Billing versus clinical versus epidemiological needs

There is an inherent tension in the use of EHR-based data for research purposes: the EHR was never originally designed for research. In the U.S., the Health Information Technology for Economic and Clinical Health Act, which promoted EHRs as a platform for comparative effectiveness research, was an attempt to address this deficiency [26]. A brief history of the evolution of the modern EHR reveals a technology that was optimized for capturing health details relevant for billing, scheduling, and clinical record keeping [27]. As such, the availability of data for fundamental markers of upstream health that are important for identifying inequities, such as socioeconomic status, race, ethnicity, and other social determinants of health (SDOH), may be insufficiently captured in the EHR [17, 18]. Similarly, behavioral risk factors, such as being a sexual minority person, have historically been insufficiently recorded as discrete variables. It is only recently that such data are beginning to be captured in the EHR [28, 29], or techniques such as NLP have made it possible to extract these details when stored in free text notes (described further in “Unstructured data: clinical notes and reports” section).

As an example, assessing clinical morbidities in the EHR may be done on the basis of extracting appropriate International Classification of Diseases (ICD) codes, used for billing and reimbursement in the U.S. These codes are known to have low sensitivity despite high specificity for accurate diagnostic status [30, 31]. Expressed as predictive values, which depend upon prevalence, presence of a diagnostic code is a likely indicator of a disease state, whereas absence of a diagnostic code is a less reliable indicator of the absence of that morbidity. There may further be variation by clinical domain in that ICD codes may exist but not be used in some specialties [32], variation by coding vocabulary such as the use of SNOMED for clinical documentation versus ICD for billing necessitating an ontology mapper [33], and variation by the use of “rule-out” diagnostic codes resulting in false-positive diagnoses [34–36]. Relatedly is the notion of upcoding, or the billing of tests, procedures, or diagnoses to receive inflated reimbursement, which, although posited to be problematic in EHRs [37] in at least one study, has not been shown to have occurred [38]. In the U.S., the billing and reimbursement model, such as fee-for-service versus managed care, may result in varying diagnostic code sensitivities and specificities, especially if upcoding is occurring [39]. In short, there is potential for misclassification of key health data in the EHR.

Misclassification can potentially be addressed through a validation study (resources permitting) or application of quantitative bias analysis, and there is a rich literature regarding the treatment of misclassified data in statistics and epidemiology. Readers are referred to these texts as a starting point [40, 41]. Duda et al. and Shepherd et al. have described an innovative data audit approach applicable to secondary analysis of observational data, such as EHR-derived data, that incorporates the audit error rate directly in the regression analysis to reduce information bias [42, 43]. Outside of methodological tricks in the face of imperfect data, researchers must proactively engage with clinical and informatics colleagues to ensure that the right data for the research interests are available and accessible.

### Sub-challenge #2.2: Consistency in data and interpretation

For the epidemiologist, abstracting data from the EHR into a research-ready analytic dataset presents a host of complications surrounding data availability, consistency and interpretation. It is easy to conflate the total volume of data in the EHR with data that are usable for research, however expectations should be tempered. Weiskopf et al. have noted such challenges for the researcher: in their study, less than 50% of patient records had “complete” data for research purposes per their four definitions of completeness [44]. Decisions made about the treatment of incomplete data can induce selection bias or impact precision of estimates (see Challenges #1, #3, and #4). The COVID-19 pandemic has further demonstrated the challenge of obtaining research data from EHRs across multiple health systems [45]. On the other hand, EHRs have a key advantage of providing near real-time data as opposed to many epidemiological studies that have a specific endpoint or are retrospective in nature. Such real-time data availability was leveraged during COVID-19 to help healthcare systems manage their pandemic response [46, 47]. Logistical and technical issues aside, healthcare and documentation practices are nuanced to their local environments. In fact, researchers have demonstrated how the same research question analyzed in distinct clinical databases can yield different results [48].

Once the data are obtained, choices regarding operationalization of variables have the potential to induce information bias. Several hypothetical examples can help demonstrate this point. As a first example, differences in laboratory reporting may result in measurement error or misclassification. While the order for a particular laboratory assay is likely consistent within the healthcare system, patients frequently have a choice where to have that order fulfilled. Given the breadth of assays and reporting differences that may differ lab to lab [49], it is possible that the researcher working with the raw data may not consider all possible permutations. In other words, there may be lack of consistency in the reporting of the assay results. As a second example, raw clinical data requires interpretation to become actionable. A researcher interested in capturing a patient’s Charlson comorbidity index, which is based on 16 potential diagnoses plus the patient’s age [50], may never find such a variable in the EHR. Rather, this would require operationalization based on the raw data, each of which may be misclassified. Use of such composite measures introduces the notion of “differential item functioning”, whereby a summary indicator of a complexly measured health phenomenon may differ from group to group [51]. In this case, as opposed to a measurement error bias, this is one of residual confounding in that a key (unmeasured) variable is driving the differences. Remediation of these threats to validity may involve validation studies to determine the accuracy of a particular classifier, sensitivity analysis employing alternative interpretations when the raw data are available, and omitting or imputing biased or latent variables [40, 41, 52]. Importantly, in all cases, the epidemiologists should work with the various health care providers and personnel who have measured and recorded the data present in the EHR, as they likely understand it best.

Furthermore and related to “Billing versus Clinical versus Epidemiological Needs” section, the healthcare system in the U.S. is fragmented with multiple payers, both public and private, potentially exacerbating the data quality issues we describe, especially when linking data across healthcare systems. Single payer systems have enabled large and near-complete population-based studies due to data availability and consistency [53–55]. Data may also be inconsistent for retrospective longitudinal studies spanning many years if there have been changes to coding standards or practices over time, for example due to the transition from ICD-9 to ICD-10 largely occurring in the mid 2010s or the adoption of the Patient Protection and Affordable Care Act in the U.S. in 2010 with its accompanying changes in billing. Exploratory data analysis may reveal unexpected differences in key variables, by place or time, and recoding, when possible, can enforce consistency.

### Sub-challenge #2.3: Unstructured data: clinical notes and reports

There may also be scenarios where structured data fields, while available, are not traditionally or consistently used within a given medical center or by a given provider. For example, reporting of adverse events of medications, disease symptoms, and vaccinations or hospitalizations occurring at different facility/health networks may not always be entered by providers in structured EHR fields. Instead, these types of patient experiences may be more likely to be documented in an unstructured clinical note, report (e.g. pathology or radiology report), or scanned document. Therefore, reliance on structured data to identify and study such issues may result in underestimation and potentially biased results.

Advances in NLP currently allow for information to be extracted from unstructured clinical notes and text fields in a reliable and accurate manner using computational methods. NLP utilizes a range of different statistical, machine learning, and linguistic techniques, and when applied to EHR data, has the potential to facilitate more accurate detection of events not traditionally located or consistently used in structured fields. Various NLP methods can be implemented in medical text analysis, ranging from simplistic and fast term recognition systems to more advanced, commercial NLP systems [56]. Several studies have successfully utilized text mining to extract information on a variety of health-related issues within clinical notes, such as opioid use [57], adverse events [58, 59], symptoms (e.g., shortness of breath, depression, pain) [60], and disease phenotype information documented in pathology or radiology reports, including cancer stage, histology, and tumor grade [61], and lupus nephritis [32]. It is worth noting that scanned documents involve an additional layer of computation, relying on techniques such as optical character recognition, before NLP can be applied.

Hybrid approaches that combine both narrative and structured data, such as ICD codes, to improve accuracy of detecting phenotypes have also demonstrated high performance. Banerji et al. found that using ICD-9 codes to identify allergic drug reactions in the EHR had a positive predictive value of 46%, while an NLP algorithm in conjunction with ICD-9 codes resulted in a positive predictive value of 86%; negative predictive value also increased in the combined algorithm (76%) compared to ICD-9 codes alone (39%) [62]. In another example, researchers found that the combination of unstructured clinical notes with structured data for prediction tasks involving in-hospital mortality and 30-day hospital readmission outperformed models using either clinical notes or structured data alone [63]. As we move forward in analyzing EHR data, it will be important to take advantage of the wealth of information buried in unstructured data to assist in phenotyping patient characteristics and outcomes, capture missing confounders used in multivariate analyses, and develop prediction models.

## Challenge #3: Missing measurements

While clinical notes may be useful to recover incomplete information from structured data fields, it may be the case that certain variables are not collected within the EHR at all. As mentioned above, it is important to remember that EHRs were not developed as a research tool (see “Billing versus clinical versus epidemiological needs” section), and important variables often used in epidemiologic research may not be typically included in EHRs including socioeconomic status (education, income, occupation) and SDOH [17, 18]. Depending upon the interest of the provider or clinical importance placed upon a given variable, this information may be included in clinical notes. While NLP could be used to capture these variables, because they may not be consistently captured, there may be bias in identifying those with a positive mention as a positive case and those with no mention as a negative case. For example, if a given provider inquires about homelessness of a patient based on knowledge of the patient’s situation or other external factors and documents this in the clinical note, we have greater assurance that this is a true positive case. However, lack of mention of homelessness in a clinical note should not be assumed as a true negative case for several reasons: not all providers may feel comfortable asking about and/or documenting homelessness, they may not deem this variable worth noting, or implicit bias among clinicians may affect what is captured. As a result, such cases (i.e. no mention of homelessness) may be incorrectly identified as “not homeless,” leading to selection bias should a researcher form a cohort exclusively of patients who are identified as homeless in the EHR.

Not adjusting for certain measurements missing from EHR data can also lead to biased results if the measurement is an important confounder. Consider the example of distinguishing between prevalent and incident cases of disease when examining associations between disease treatments and patient outcomes [64]. The first date of an ICD code entered for a given patient may not necessarily be the true date of diagnosis, but rather documentation of an existing diagnosis. This limits the ability to adjust for disease duration, which may be an important confounder in studies comparing various treatments with patient outcomes over time, and may also lead to reverse causality if disease sequalae are assumed to be risk factors.

Methods to supplement EHR data with external data have been used to capture missing information. These methods may include imputation if information (e.g. race, lab values) is collected on a subset of patients within the EHR. It is important to examine whether missingness occurs completely at random or at random (“ignorable”), or not at random (“non-ignorable”), using the data available to determine factors associated with missingness, which will also inform the best imputation strategy to pursue, if any [65, 66]. As an example, suppose we are interested in ascertaining a patient's BMI from the EHR. If men were less likely to have BMI measured than women, the probability of missing data (BMI) depends on the observed data (gender) and may therefore be predictable and imputable. On the other hand, suppose underweight individuals were less likely to have BMI measured; the probability of missing data depends on its own value, and as such is non-predictable and may require a validation study to confirm. Alternatively to imputing missing data, surrogate measures may be used, such as inferring area-based SES indicators, including median household income, percent poverty, or area deprivation index, by zip code [67, 68]. Lastly, validation studies utilizing external datasets may prove helpful, such as supplementing EHR data with claims data that may be available for a subset of patients (see Challenge #4).

As EHRs are increasingly being used for research, there are active pushes to include more structured data fields that are important to population health research, such as SDOH [69]. Inclusion of such factors are likely to result in improved patient care and outcomes, through increased precision in disease diagnosis, more effective shared decision making, identification of risk factors, and tailoring services to a given population’s needs [70]. In fact, a recent review found that when individual level SDOH were included in predictive modeling, they overwhelmingly improved performance in medication adherence, risk of hospitalization, 30-day rehospitalizations, suicide attempts, and other healthcare services [71]. Whether or not these fields will be utilized after their inclusion in the EHR may ultimately depend upon federal and state incentives, as well as support from local stakeholders, and this does not address historic, retrospective analyses of these data.

## Challenge #4: Missing visits

Beyond missing variable data that may not be captured during a clinical encounter, either through structured data or clinical notes, there also may be missing information for a patient as a whole. This can occur in a variety of ways; for example, a patient may have one or two documented visits in the EHR and then is never seen again (i.e. right censoring due to lost to follow-up), or a patient is referred from elsewhere to seek specialty care, with no information captured regarding other external issues (i.e. left censoring). This may be especially common in circumstances where a given EHR is more likely to capture specialty clinics versus primary care (see Challenge #1). A third scenario may include patients who appear, then are not observed for a long period of time, and then reappear: this case is particularly problematic as it may appear the patient was never lost to follow up but simply had fewer visits. In any of these scenarios, a researcher will lack a holistic view of the patient’s experiences, diagnoses, results, and more. As discussed above, assuming absence of a diagnostic code as absence of disease may lead to information and/or selection bias. Further, it has been demonstrated that one key source of bias in EHRs is “informed presence” bias, where those with more medical encounters are more likely to be diagnosed with various conditions (similar to Berkson’s bias) [72].

Several solutions to these issues have been proposed. For example, it is common for EHR studies to condition on observation time (i.e. ≥n visits required to be eligible into cohort); however, this may exclude a substantial amount of patients with certain characteristics, incurring a selection bias or limiting the generalizability of study findings (see Challenge #1). Other strategies attempt to account for missing visit biases through longitudinal imputation approaches; for example, if a patient missed a visit, a disease activity score can be imputed for that point in time, given other data points [73, 74]. Surrogate measures may also be used to infer patient outcomes, such as controlling for “informative” missingness as an indicator variable or using actual number of missed visits that were scheduled as a proxy for external circumstances influencing care [20]. To address “informed presence” bias described above, conditioning on the number of health-care encounters may be appropriate [72]. Understanding the reason for the missing visit may help identify the best course of action and before imputing, one should be able to identify the type of missingness, whether “informative” or not [65, 66]. For example, if distance to a healthcare location is related to appointment attendance, being able to account for this in analysis would be important: researchers have shown how the catchment of a healthcare facility can induce selection bias [21]. Relatedly, as telehealth becomes more common fueled by the COVID-19 pandemic [75, 76], virtual visits may generate missingness of data recorded in the presence of a provider (e.g., blood pressure if the patient does not have access to a sphygmomanometer; see Challenge #3), or necessitate a stratified analysis by visit type to assess for effect modification.

Another common approach is to supplement EHR information with external data sources, such as insurance claims data, when available. Unlike a given EHR, claims data are able to capture a patient’s interaction with the health care system across organizations, and additionally includes pharmacy data such as if a prescription was filled or refilled. Often researchers examine a subset of patients eligible for Medicaid/Medicare and compare what is documented in claims with information available in the EHR [77]. That is, are there additional medications, diagnoses, hospitalizations found in the claims dataset that were not present in the EHR. In a study by Franklin et al., researchers utilized a linked database of Medicare Advantage claims and comprehensive EHR data from a multi-specialty outpatient practice to determine which dataset would be more accurate in predicting medication adherence [77]. They found that both datasets were comparable in identifying those with poor adherence, though each dataset incorporated different variables.

While validation studies such as those using claims data allow researchers to gain an understanding as to how accurate and complete a given EHR is, this may only be limited to the specific subpopulation examined (i.e. those eligible for Medicaid, or those over 65 years for Medicare). One study examined congruence between EHR of a community health center and Medicaid claims with respect to diabetes [78]. They found that patients who were older, male, Spanish-speaking, above the federal poverty level, or who had discontinuous insurance were more likely to have services documented in the EHR as compared to Medicaid claims data. Therefore, while claims data may help supplement and validate information in the EHR, on their own they may underestimate care in certain populations.

## Discussion

Research utilizing EHR data has undoubtedly positively impacted the field of public health through its ability to provide large-scale, longitudinal data on a diverse set of patients, and will continue to do so in the future as more epidemiologists take advantage of this data source. EHR data’s ability to capture individuals that traditionally aren’t included in clinical trials, cohort studies, and even claims datasets allows researchers to measure longitudinal outcomes in patients and perhaps change the understanding of potential risk factors.

However, as outlined in this review, there are important caveats to EHR analysis that need to be taken into account; failure to do so may threaten study validity. The representativeness of EHR data depends on the catchment area of the center and corresponding target population. Tools are available to evaluate and remedy these issues, which are critical to study validity as well as extrapolation of study findings. Data availability and interpretation, missing measurements, and missing visits are also key challenges, as EHRs were not specifically developed for research purposes, despite their common use for such. Taking advantage of all available EHR data, whether it be structured or unstructured fields through NLP, will be important in understanding the patient experience and identifying key phenotypes. Beyond methods to address these concerns, it will remain crucial for epidemiologists and data analysts to engage with clinicians and informaticians at their institutions to ensure data quality and accessibility by forming multidisciplinary teams around specific research projects. Lastly, integration across multiple EHRs, or datasets that encompass multi-institutional EHR records, add an additional layer of data quality and validity issues, with the potential to exacerbate the above-stated challenges found within a single EHR. At minimum, such studies should account for correlated errors [79, 80], and investigate whether modularization, or submechanisms that determine whether data are observed or missing in each EHR, exist [65].

The identified challenges may also apply to secondary analysis of other large healthcare databases, such as claims data, although it is important not to conflate the two types of data. EHR data are driven by clinical care and claims data are driven by the reimbursement process where there is a financial incentive to capture diagnoses, procedures, and medications [48]. The source of data likely influences the availability, accuracy, and completeness of data. The fundamental representation of data may also differ as a record in a claims database corresponds to a “claim” as opposed to an “encounter” in the EHR. As such, the representativeness of the database populations, the sensitivity and specificity of variables, as well as the mechanisms of missingness in claims data may differ from EHR data. One study that evaluated pediatric quality care measures, such as BMI, noted inferior sensitivity based on claims data alone [81]. Linking claims data to EHR data has been proposed to enhance study validity, but many of the caveats raised in herein still apply [82].

Although we focused on epidemiological challenges related to study validity, there are other important considerations for researchers working with EHR data. Privacy and security of data as well as institutional review board (IRB) or ethics board oversight of EHR-based studies should not be taken for granted. For researchers in the U.S., Goldstein and Sarwate described Health Insurance Portability and Accountability Act (HIPAA)-compliant approaches to ensure the privacy and security of EHR data used in epidemiological research, and presented emerging approaches to analyses that separate the data from analysis [83]. The IRB oversees the data collection process for EHR-based research and through the HIPAA Privacy Rule these data typically do not require informed consent provided they are retrospective and reside at the EHR’s institution [84]. Such research will also likely receive an exempt IRB review provided subjects are non-identifiable.

## Conclusions

As EHRs are increasingly being used for research, epidemiologists can take advantage of the many tools and methods that already exist and apply them to the key challenges described above. By being aware of the limitations that the data present and proactively addressing them, EHR studies will be more robust, informative, and important to the understanding of health and disease in the population.

## Data Availability

All data and materials used in this review are described herein.

## Abbreviations

BMI: Body Mass Index
EHR: Electronic Health Record
ICD: International Classification of Diseases
IRB: Institutional review board/ethics board
HIPAA: Health Insurance Portability and Accountability Act
NLP: Natural Language Processing
SDOH: Social Determinants of Health
SES: Socioeconomic Status
